# Improving Encapsulation of Hydrophilic Chloroquine Diphosphate into Biodegradable Nanoparticles: A Promising Approach against Herpes Virus Simplex-1 Infection

**DOI:** 10.3390/pharmaceutics10040255

**Published:** 2018-12-03

**Authors:** Tábata Loíse Cunha Lima, Renata de Carvalho Feitosa, Emanuell dos Santos-Silva, Alaine Maria dos Santos-Silva, Emerson Michell da Silva Siqueira, Paula Renata Lima Machado, Alianda Maira Cornélio, Eryvaldo Sócrates Tabosa do Egito, Matheus de Freitas Fernandes-Pedrosa, Kleber Juvenal Silva Farias, Arnóbio Antônio da Silva-Júnior

**Affiliations:** 1 Laboratory of Pharmaceutical Technology and Biotechnology, Department of Pharmacy, Federal University of Rio Grande do Norte, UFRN, Gal. Gustavo Cordeiro de Farias, Petrópolis, Natal 59.072-570, Brazil; tabata.cunhalima@gmail.com (T.L.C.L.); rcarvalhofeitosa@gmail.com (R.d.C.F.); emanuell14@gmail.com (E.d.S.-S.); alainemaria@ufrn.edu.br (A.M.d.S.-S.); mffpedrosa@gmail.com (M.d.F.F.-P.); 2 Graduate Program in Health Sciences, Federal University of Rio Grande do Norte (UFRN), Natal 59.072-570, Brazil; socratesegito@gmail.com; 3 Graduate Program in Development and Technological Innovation in Medicines, Federal University of Rio Grande do Norte (UFRN), Natal 59.072-570, Brazil; siqueira.emerson.emss@gmail.com; 4 Department of Clinical Analysis and Toxicology, Federal University of Rio Grande do Norte, UFRN, Gal. Gustavo Cordeiro de Farias, Petrópolis, Natal 59.072-570, Brazil; paulamachado@ufrnet.br; 5 Department of Morphology, Federal University of Rio Grande do Norte, UFRN, Natal 59.072-570, Brazil; aliandamaira@gmail.com

**Keywords:** nanoparticles, poly(lactic acid), chloroquine, antiviral activity, herpes simplex, biological barriers

## Abstract

Chloroquine diphosphate (CQ) is a hydrophilic drug with low entrapment efficiency in hydrophobic nanoparticles (NP). Herpes simplex virus type 1 (HSV-1) is an enveloped double-stranded DNA virus worldwide known as a common human pathogen. This study aims to develop chloroquine-loaded poly(lactic acid) (PLA) nanoparticles (CQ-NP) to improve the chloroquine anti- HSV-1 efficacy. CQ-NP were successfully prepared using a modified emulsification-solvent evaporation method. Physicochemical properties of the NP were monitored using dynamic light scattering, atomic force microscopy, drug loading efficiency, and drug release studies. Spherical nanoparticles were produced with modal diameter of <300 nm, zeta potential of −20 mv and encapsulation efficiency of 64.1%. In vitro assays of CQ-NP performed in Vero E6 cells, using the MTT-assay, revealed different cytotoxicity levels. Blank nanoparticles (B-NP) were biocompatible. Finally, the antiviral activity tested by the plaque reduction assay revealed greater efficacy for CQ-NP compared to CQ at concentrations equal to or lower than 20 µg mL^−1^ (*p* < 0.001). On the other hand, the B-NP had no antiviral activity. The CQ-NP has shown feasible properties and great potential to improve the antiviral activity of drugs.

## 1. Introduction

Herpes simplex virus type 1 (HSV-1) is an enveloped double-stranded DNA virus belonging to the *Herpesviridae* family. This virus, a common human pathogen, is the main cause of oral infection, affecting the mouth and lips. In addition, HSV-1 can be the infectious agent of several diseases including recurrent cold sores, keratoconjunctivitis, and life-threatening herpes encephalitis [1,2,3]. Following primary infection, HSV establishes infections in the neurons of the sensory ganglia from where it may become latent in the body [4,5]. In the nervous system, the virus is protected from the host immune system as a latent state, which can be reactivated by several factors, such as hormonal changes, ultraviolet (UV) light, and mainly the stress [6]. The treatment of infection caused by the HSV-1 include the use of drugs, such as acyclovir, valacyclovir, famciclovir, penciclovir, and cidofovirare [7]. Some pharmacokinetic limitations of these drugs can be difficult because of their transport to affected tissues, inducing non-therapeutic drug levels. Moreover, long-term of these treatments may lead to the selection of resistant HSV, being an additional concern mainly for the immunocompromised patients, which has emphasized the need for alternative strategies [8,9].

Chloroquine diphosphate (CQ) is an antimalarial drug that has been used as the first-line treatment against *Plasmodium vivax, Plasmodium ovale, Plasmodium malariae,* and *Plasmodium falciparum* infections. In addition to the antimalarial activity, CQ has been used to treat chronic diseases such as rheumatoid arthritis and systemic lupus erythematosus [10,11,12]. Recently, this drug has also attracted attention due to its anticancer [13,14,15,16,17] and antiviral activity [18,19,20,21,22,23,24]. In this context, CQ is a hydrophilic drug with low toxicity that has shown some limitations to reach an effective intracellular concentration. One of the main disadvantages of the conventional malaria treatment is the non-specific targeting to intracellular parasites, which leads to high dose administration, resulting in toxicity [25].

As expected for antimalarial drugs, the efficacy of antiviral drugs such as CQ depends on their cellular uptake. In attempt to solve this limitation of CQ against HSV-1, the nanodrug delivery systems (NDDS) are known to change drug biodistribution, decrease toxicity, modify drug release rate, and mainly, drug targeting to affected tissues/cell [26]. Among the NDDS, polymeric nanoparticles (NP) is a promising approach to improve drug uptake for a specific cell line. Their advantages include higher stability in biological fluids and easy storage conditions [27,28]. Polyesters, such as poly(lactic acid) (PLA) are biocompatible and biodegradable polymers, which are completely eliminated from the body by natural metabolic pathways. Furthermore, PLA is a polymer approved by the Food and Drug Administration (FDA) that has been widely used as a biomaterial for the preparation of nanoparticles for drug delivery [29].

Several methods have been applied to prepare NP. They can be divided into two categories: Those based on the dispersion of preformed polymers and those based on the polymerization of monomers [27]. Considering the dispersion of preformed polymers, the solvent evaporation method or the spontaneous emulsification/solvent diffusion method is a well-consolidated one [30]. Nanoprecipitation (solvent displacement method) is a one-step procedure controlled by the interfacial deposition of the polymer after its displacement from organic to aqueous phase. Due to the fast spontaneous diffusion, nanoparticle formation occurs instantaneously. Emulsification-solvent evaporation method is a two-step procedure in which the organic solution is emulsified in the aqueous phase using a high-speed homogenization or ultrasonication. The formation of NP occurs due to the polymer precipitation after the solvent evaporation [27].

The entrapment of hydrophilic drugs inside hydrophobic polymeric nanoparticles is not an easy task, especially when using the nanoprecipitation method [31,32]. Indeed, hydrophilic drugs have weak interactions with polyesters, such as PLA. Thus, the drug transport from the organic phase to the outer aqueous phase induces low entrapment efficiency. This phenomenon justifies drawbacks of less-well protected drug from degradation and a faster drug release [33]. Some strategies have been used to improve the encapsulation efficiency of hydrophilic molecules in polyester nanoparticles. Controlling pH of aqueous phase, changes in the solvent phase, use of high drug concentration, and salt addition in the aqueous phase are some examples [30].

In the context of anti- HSV-1 activity of CQ [34,35,36,37], a specific study reported by Singh et al. (1996) demonstrated the possible mechanism of action of CQ as a promising antiviral drug [38]. This drug has a character of weak base that contributes to increase the intracellular pH, affecting the virus life cycle. In addition. CQ impairs the gD protein transport from trans-Golgi network to cell membrane, which induces the formation of defective viral particles [38]. Thus, this study was performed to improve the encapsulation efficiency of hydrophilic CQ into biocompatible PLA nanoparticles, and to evaluate how the modulation of the drug release affects its anti-HSV activity. The experimental design included different preparation methods, variation in the polymer/drug ratio, and specific parameters such as pH of aqueous phase. The anti-HSV performance was assessed using Vero E6 cells.

## 2. Materials and Methods

### 2.1. Materials

Chloroquine Diphosphate (CQ), Poloxamer 407 (Pluronic^®^ F-127), and Poloxamer 188 (Pluronic^®^ F-68) were purchased from Sigma-Aldrich (Saint Louis, MO, USA). d,l-poly(lactic acid) (PLA) (inherent viscosity 0.67 dL g^−1^ at 25 °C) was purchased from LACTEL Absorbable Polymers^®^ (Birmingham, AL, USA). Dichloromethane (DCM) (dielectric constant (ε) of 9.1), ethanol (EtOH) (ε of 24.6), acetone (ACE) (ε of 20.6) and *N*-Methyl-2-pyrrolidone (NMP) were purchased from Labsynth^®^ (São Paulo, Brazil). The purified water (1.3 µS cm^−1^) was prepared from reverse osmosis purification equipment, model OS50 LX, Gehaka (Sao Paulo, Brazil). All other reagents have analytical grade.

### 2.2. Nanoprecipitation

CQ-loaded PLA nanoparticles were prepared using the adjusted nanoprecipitation method [39]. Briefly, the CQ was dissolved in 1.8 mL of a mixture of NMP:water (1:5 *v*/*v*) and added to 4.2 mL of a mixture of ACE:EtOH (1:1 *v*/*v*) containing the dissolved PLA. This solution (6 mL) was injected at an output flux of 3.0 mL min^−1^ into the aqueous phase (14 mL) containing the surfactant, poloxamer 188 (1% *w*/*v*), under magnetic stirring at 720 rpm at 25 °C. Both aqueous and organic phase were previously filtered using a 0.45 μm membrane (Sartorius^®^). The organic solvent evaporation occurred under magnetic stirring at 720 rpm, at 25 °C, overnight. Samples were stored in hermetically sealed glass flasks^,^ at 8 °C and have the size and zeta potential measured after 24 h. The Blank nanoparticles were prepared carrying out the same procedure without the addition of CQ. All experiments were performed in triplicate, and the data were expressed as mean ± standard deviation (SD).

#### 2.2.1. Effect of Drug/Polymer Ratio

Solutions containing PLA at different concentrations (0.25%, 0.50%, and 0.75% *w*/*v*) were injected in the aqueous phase containing poloxamer 188 at 1% *w*/*v*, under magnetic stirring at 720 rpm. The drug concentration was fixed in this experiment, resulting in drug/polymer ratios (CQ:PLA) of 1:5, 1:10 and 1:15 *w*/*w*.

#### 2.2.2. pH Changes in the Aqueous Phase

Two different alkalinizing agents were tested to modify the pH of the outer phase. CQ and PLA concentration were fixed at 0.05% and 0.5% *w*/*v,* respectively in the organic phase (1:10 *w*/*v*), while poloxamer 188 concentration remained fixed at 1.0% *w*/*v* in the aqueous phase. The first aqueous phase has the pH adjusted to 11.0, using 0.1 M sodium hydroxide (NaOH). The second tested aqueous phase was produced by adding 0.5 M sodium bicarbonate (NaHCO_3_) with pH adjusted to 8.4.

### 2.3. Emulsification with Solvent Evaporation

Nanoparticles were also prepared using the adjusted emulsification-solvent evaporation method [40]. Briefly, a small-scale liquid-liquid partition procedure was performed mixing 0.4 mL of 0.5 M NaHCO_3_ aqueous solution (pH = 8.4) (0.4 mL) containing CQ at 1.25% *w*/*v* with 10 mL of organic phase (DCM) containing the PLA (0.25% *w*/*w*), to obtain CQ:PLA ratio of 1:5 *w*/*w*. The flask was stirred by vortex for 3 cycles of 10 seconds each, with intervals of 1 min between each cycle. After the partitioning step, the supernatant (NaHCO_3_ solution) was removed and 4 mL of the organic phase containing CQ and PLA was taken and injected at 1.0 mL min^−1^ into the aqueous phase (16 mL) containing the surfactant, poloxamer 407 (0.75% *w*/*v*), under magnetic stirring at 720 rpm, at 25 °C. The organic and aqueous phases were previously filtered using 0.45 μm membranes. Emulsification was produced by using Ultra-turrax equipment stirring (IKA Labortechnik, Germany) for 18 min and evaporation of the solvent at 25 °C, under magnetic stirring at 750 rpm, overnight. Samples were stored in hermetically sealed glass flasks at 8 °C and have the size and zeta potential measured after 24 h. All experiments were performed in triplicate, and the data are expressed as mean ± standard deviation (SD). The amount of drug at organic phase was analytically determined by UV spectrophotometry at 330 nm. The blank nanoparticles were prepared using the same procedure without the addition of CQ in the buffered solution. All experiments were performed in triplicate, and the data were expressed as mean ± standard deviation (SD).

### 2.4. Particle Size and Zeta Potential Measurements

Mean particle size and polydispersity index (PdI) were assessed by using Dynamic Light Scattered (DLS) in a particle size analyzer (Brookhaven Instruments, Holtsville, NY, US), at 659 nm wavelength, 90° detection angle, and at 25 °C. Zeta potential (ζ potential) measurements were performed in the same equipment applying a field strength of about 5.9 V·cm^−1^ by using the electrophoretic mobility. The measurements were performed for at least ten determinations for each sample diluted at 1:50 (*v*/*v*) with purified water. All experiments were performed in triplicate and data were expressed as mean ± standard deviation (SD).

### 2.5. Drug-Loading Efficiency

Samples were centrifuged at 16,000× *g* for 60min. at 4 °C using the ultra-centrifugal filter (Sartorius^®^, Vivaspin 2, Ultra-15MWCO 10 kDa). Drug concentration at the supernatant was assessed using UV-Vis Spectrophometry method at 330 nm (Thermo Fisher Scientific, 60S Evolution, Madison, WI, US). All analyses were performed in triplicate, and the data were expressed as mean ± standard deviation (SD). The encapsulation efficiency (EE) was calculated by using the Equation (1), where [Drug]_total_ is the total drug amount added and [Drug]_free_ is the non-entrapped drug in supernatant after centrifugation.

(1) $$\mathrm{EE}\;\left(\%\right)\;=\frac{{\left[\mathrm{Drug}\right]}_{\mathrm{total}}-{\left[\mathrm{Drug}\right]}_{\mathrm{free}}}{{\left[\mathrm{Drug}\right]}_{\mathrm{total}}}\;\times \;100$$

### 2.6. Atomic Force Microscopy (AFM)

Shape and surface of the drug-free and CQ-NP were observed by using 2D and 3D AFM images. The dispersions were dropped in a cover slip, dried under desiccator for 24 h, and then, analyzed in an AFM, SPM-9700, Shimadzu (Tokyo, Japan), at room temperature with a cantilever non-contact, 1 Hz scanning.

### 2.7. In Vitro Drug Release

The drug release profile of CQ-NP was assessed through the in vitro assay using static Franz vertical diffusion cells (Crown Scientific, Sommerville, MA, US), thermostatized at 37 ± 0.5 °C. In the hermetically sealed donor compartment, 1.0 mL of formulations were applied and separated from receptor compartment using dialysis membrane with cut-off 12–14 kDa (Sigma-Aldrich), previously hydrated in phosphate buffer (pH = 7.4) for 24 h. The receptor compartment filled with 11.0 mL of buffer phosphate solution, adjusted to pH 7.4, remained under magnetic stirring at 360 rpm for all experiments. At specific intervals, aliquots of 1.0 mL were taken for UV spectrophotometry analysis at 330 nm, previously validated. The same volume of freshly buffer solution replaced the medium to maintain the sink conditions. The experiment was also performed for free CQ in aqueous solution, as control. All analyses were performed in triplicate and data were expressed as mean ± standard deviation (SD).

### 2.8. Cells and Viruses

Vero E6 cells (continuous cell lineage originated from the kidney of African green monkeys), were kindly donated by the Department of Internal Medicine, School of Medicine of Ribeirão Preto—University of Sao Paulo-USP, Brazil. The cells were maintained at 37 °C and 5% CO_2_ and cultured in Leibovitz-15 culture medium (L-15) with l-glutamine (Invitrogen, New York, NY, US) supplemented with 2% or 10% of fetal bovine serum (FBS), 1% of antibiotic-antimycotic solution 100X (Gibco^®^), and 10% of triptose phosphate [19,20].

The virus HSV-1 (strain KOS) was kindly provided by Dra. Cláudia Maria Oliveira Simões and Msc. Laurita Boff, from the College of Pharmacy, Federal University of Santa Catarina, Brazil. The virus was propagated and titrated by plaque-forming units assay (PFU) on Vero E6 cells [41]. Viral stocks were stored at −80 °C until use.

### 2.9. Cell Viability Studies

The viability of Vero cells following exposure for 24–48 h at different concentrations of CQ, CQ-NP and blank nanoparticles (B-NP) was assessed using the 3-(4,5-dimethylthiazol-2-yl)-2,5-diphenyltetrazolium bromide (MTT) method. Briefly, Vero cells (2 × 10^4^ cells/well) were cultured in 96-well plates in L-15 medium with 10% of FBS and maintained at 37 °C and 5% CO_2_. After 24 h, the cells were exposed to decreasing concentrations (30, 20, 10, 5, and 2.5 µg mL^−1^) of CQ and a two-fold serial dilution of nanoparticles (range 500–7.81 µg mL^−1^) solution suspended in medium supplemented with 2% of FBS. Before the cytotoxicity assay, CQ dissolved in L-15 at 2% were filtered through a 0.22 μm sterile filter (Sartorius ^®^), and CQ-NP and B-NP were filtered through a 0.45 μm sterile filter (Sartorius ^®^). After 24 and 48 h of incubation, medium was collected and 50 µL of MTT (Sigma, Germany, ref.: M2128-1G) solution (1 mg mL^−1^) was added per well. Plates were, then, incubated for 4 h at 37 °C and 5% CO_2_. Posteriorly, 100 µL of DMSO was added in each well and the absorbance was measured at 540 nm in a microplate reader (Biotek^®^, Epoch model). All measurements were carried out in triplicate (three plates) with three replicates for each dilution. The cell viability was defined in comparison to untreated controls. The relative cell viability (%) in the sample treated wells with respect to the control wells was estimated by the Equation (2):

(2) $$\%\;\mathrm{Cell}\;\mathrm{Viability}=\left[\frac{\left(A\right)\;\mathrm{tested}}{\left(B\right)\;\mathrm{control}\;}\right]\;\times \;100$$

where (A) tested and (B) control are the absorbance of treated sample and control sample, respectively. All experiments were set up in triplicates.

For the determination of the cytotoxic concentration (CC_50_) values, nonlinear regression of concentration-response curves was used. The CC_50_ was defined as the concentration that reduced cell viability by 50% when compared to untreated controls.

### 2.10. Cell Infection

For this assay, the titer of the virus preparation was 3.75 × 10^7^ PFU/mL. For the plaque reduction assay, Vero cell monolayers were infected with 100 PFU/well of the HSV-1 for 1 h at 37 °C. The inoculum with non-attached viral particles was removed and the cells were washed with PBS. Finally, the infected cells were treated according to the assay.

### 2.11. Antiviral Activity

The CQ, CQ-NP, and B-NP were tested for antiviral activity against HSV-1 by the plaque reduction assay. This assay was performed according to the standard method described Caldas dos Santos, 2017 [41] and Beram-Pinto, 2009 [42]. Vero cells were seeded in 24-well plates (2 × 10^5^ cells/well) and infected with HSV-1 diluted in medium without supplement (100 PFU/well) for 1 h at 37 °C, except for the negative controls. After incubation, the viral inoculum was removed and the monolayers were washed with PBS. Subsequently, L-15 medium containing 3% of carboxymethyl-cellulose (CMC; Saint Louis, MO, USA) (overlay) was added and plates incubated at 37 °C for 48 h and overlaid, in duplicate, with L-15 containing 2% of CMC in the presence or absence of different non-cytotoxic concentrations (30, 20, 10, 5, and 2.5 µg mL^−1^) of the test compound. The plates were, then, incubated for 48 h at 37 °C. After this period, the medium in the wells was removed and the cells were stained with naphthol blue-black (Sigma). The number of plaques in each well were then counted. All measurements were carried out in duplicate (two plates) with three replicates for each dilution. The percent of inhibitory of any concentration was determined using the Equation (3).

(3) $$\%\;\mathrm{of}\;\mathrm{inhibition}=\left[1-\frac{\left(\mathrm{number}\;\mathrm{of}\;\mathrm{plaque}\right)\;\mathrm{tested}}{\mathrm{Number}\;\mathrm{of}\;\mathrm{viral}\;\mathrm{control}\;\mathrm{plaque}\;}\right]\;\times \;100$$

The concentration of treatments that reduced viral replication by 50% (IC_50_) was calculated by regression analysis of the dose-response curves generated from the data. Viral controls were considered with 0% of inhibition because they are not receiving any treatment. The ratio between CC_50_ and IC_50_ values was calculated to obtain the selectivity index (SI) of each sample. Acyclovir (ACV; Pharma nostra; China) was used as positive control.

### 2.12. Statistical

All experimental values were expressed as mean ± standard deviation (SD). The pairwise comparisons of the analytical data were performed using the Student’s t-test. For the antiviral assay and cell viability, the multiple comparisons were performed using analysis of variance (ANOVA) at *p* = 0.05 significance level, followed by Bonferroni’s test and Dunnett’s test [43,44].

## 3. Results

### 3.1. Preparation of Drug-Loaded Nanoparticles by the Nanoprecipitation Method

Small CQ-NP were successfully prepared using the parameters selected for the nanoprecipitation method. For each experiment B-NP were produced using the same experimental parameters in absence of drug. The NP <250 nm with negative zeta potential and narrow particle size distribution (PdI < 0.2) were identified for tested formulations (Table 1). Three different CQ:PLA ratios were tested (Formulations 1–3) using the same slightly acid aqueous phase (pH = 6.4). However, EE% levels less than 11% were identified for these samples, which decreased according to the CQ: PLA ratio (from 10.6% to 3.4%). In addition, the zeta potential becomes more negative (from −3.13 mV to −14.42 mV). Due to the pH-dependent solubility character of the CQ, two alkaline aqueous phases were tested aiming to improve the EE% (Formulations 4 and 5) (Table 1). Using 0.1 M NaOH as alkalizing agent (Formulation 4), it was possible to achieve an EE% of about 11.4%. On the other hand, the Formulation 5, produced using 0.5 M NaHCO_3_, induced an EE% of about 25%.

### 3.2. Preparation of Drug-Loaded Nanoparticles by the Emulsification-Solvent Evaporation Method

In this approach, an adjusted emulsification-solvent evaporation method was used, applying a small-scale liquid-liquid partition to provide the maximum concentration of chloroquine free base (CQ-fb) in the organic phase. All experiments have the CQ-fb amount in organic phase analytically controlled using UV spectrophotometry. An average level of 97.51% of CQ-fb dissolved in the DCM was achieved. NP < 300 nm with negative zeta potential and PdI of about 0.3 were produced using the selected parameters (Table 2). The strategy to induce CQ-fb in an immiscible organic phase structured as droplets dispersed in an alkaline 0.5M NaHCO_3_ aqueous solution (pH = 8.4) provided an excellent EE% of 64.1%. This formulation (formulation 6) was then selected for further studies. 

### 3.3. Morphology

**Figure 1:**
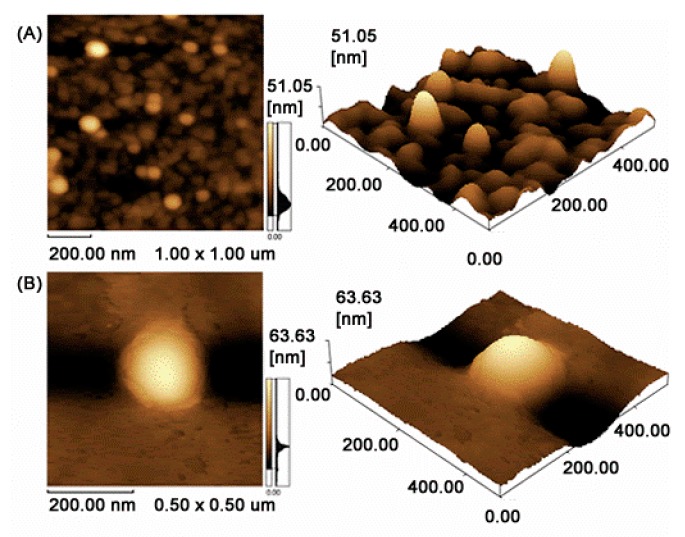
AFM images of (**A**) blank nanoparticles and (**B**) drug-loaded nanoparticles in 2D and 3D, respectively.

The shape and surface of B-NP and CQ-NP (formulation 6) produced by the emulsification-solvent evaporation method were assessed using 2D and 3D Atomic Force Microscopy (AFM) images (Figure 1). Both nanoparticle formulations have shown spherical shape and slight smooth surface.

### 3.4. In Vitro Drug Release

**Figure 2:**
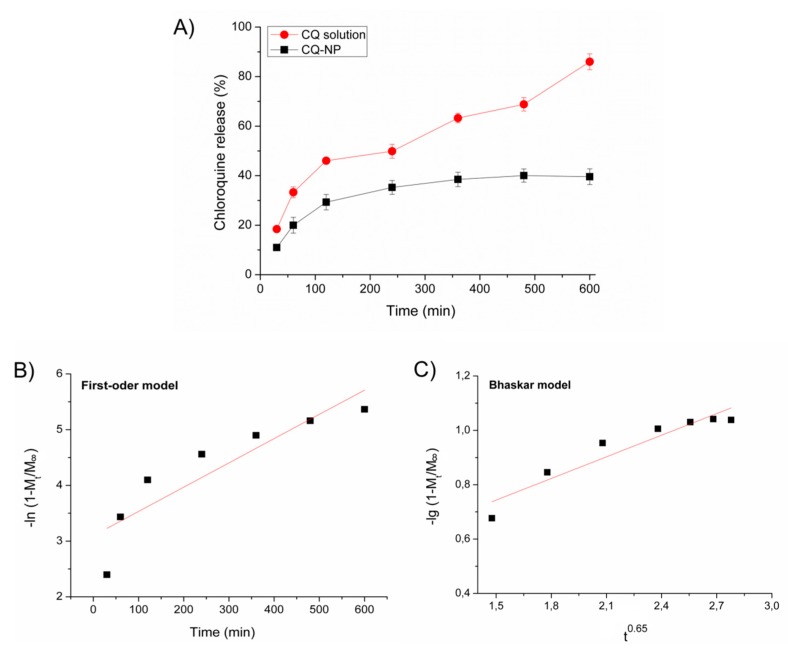

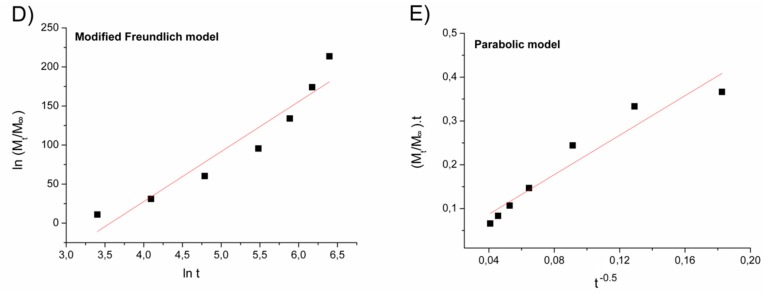
Experimental in vitro release profile of (**A**) chloroquine (CQ) solution (●) and chloroquine-loaded nanoparticles (CQ-NP) (■) from different samples and respective mathematical modeling adjustment of nanoparticles of data using: (**B**) first-order model; (**C**) Bhaskar model; (**D**) modified Freundlich model; and (**E**) Parabolic model. Notes: The data are expressed as mean ± standard deviation (SD) (*n* = 2).

The drug release experiment was performed for the formulation 6, produced by emulsification with solvent evaporation method. Its drug release profile is shown in Figure 2. The CQ-loaded NP exhibited the desired slow drug release profile, compared to the free CQ drug aqueous solution.

Data were subjected to four different diffusion kinetic linear models, which include the first-order model (Figure 2B), the Bhaskar model (Figure 2C), the Freundlich model (Figure 2D), and the parabolic model (Figure 2E). The drug release rate constant (*k*) and correlation coefficients (*R*) from distinct kinetic models are shown in the Table 2. The parabolic diffusion model fitted the CQ release from PLA nanoparticles better than the others mathematical models, giving a linear correlation coefficient of 0.95.

### 3.5. Cell Viability Studies

**Figure 3:**
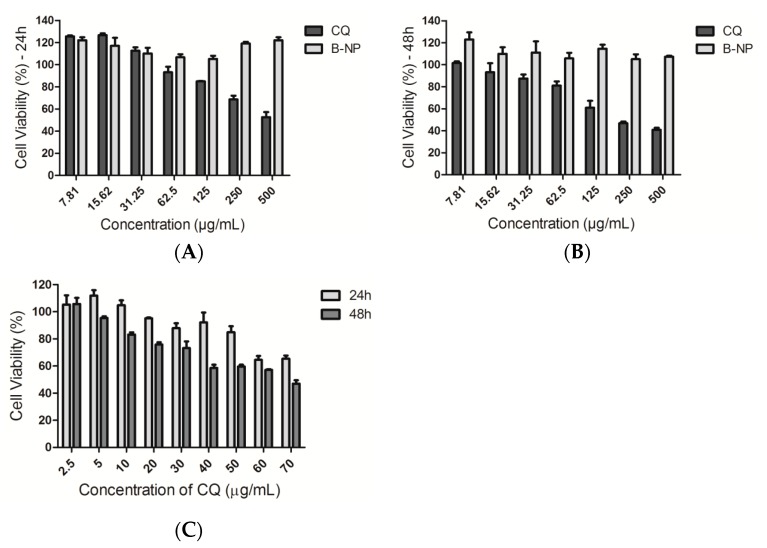
Cell viability in Vero E6 cells. Assay performed after the incubation of CQ and B-NP samples after (**A**) 24 h and (**B**) 48 h of incubation, respectively; and (**C**) assay performed after 24 h and 48 h of incubation for CQ-NP sample. Concentrations lower than 62.5 µg mL^−1^ for CQ, and 30 µg mL^−1^ for CQ-NP exhibited low cell cytotoxicity when compared to untreated controls. On the other hand, all tested concentrations for B-NP did not induce significant cytotoxicity.

Before the experiments of the antiviral activity, the cytotoxicity of CQ, CQ-NP (formulation 6) and B-NP in Vero E6 cells using the MTT assay was evaluated (Figure 3). The B-NP have shown no cytotoxic effect at the concentration range from seven to 500 µg mL^−1^, for 24 h and 48 h (Figure 3A,B). On the other hand, the drug (CQ) exhibited a cytotoxic concentration-dependent profile, which was time-dependent (Figure 3A,B). In addition, the serial dilution (500–7.81 µg mL^−1^) suggested no significant cytotoxicity to treated cells with these formulations at concentrations ≤ 62.5 µg mL^−1^, for 48 h. 

The experiment with CQ-NP considered a serial dilution (70–2.5 µg mL^−1^) to evaluate the effect of NP on the drug toxicity at the same conditions of the anterior experiments (Figure 3C). The concentration of 70 µg mL^−1^ was used as starting point for serial dilution, once cell viability above 80% was achieved after the treatment with a CQ at 62.5 µg mL^−1^. As observed for CQ (Figure 3A,B), the CQ-NP samples also exhibited a concentration and time-pendent cytotoxicity profile (Figure 3C). The NP increased the cytotoxic effect of the drug at the similar concentration range, suggesting greater drug uptake by the cells. The average CC_50_ identified for free-CQ drug was more threefold (222.6 µg mL^−1^) than that for CQ-NP (67.9 µg mL^−1^) (Table 3). In addition, the cell viability ≥ 80% was observed just for concentration levels lower than 30 µg mL^-1^. This achievement was fundamental to choose the concentration range (30 to 2.5 µg mL^−1^) to be used in further in vitro infectious assays for B-NP, CQ, and CQ-NP. 

### 3.6. Antiviral Activity

The HSV-1 activity of B-NP, CQ, and CQ-NP (formulation 6) in the infected Vero E6 cells were assessed using the standard plaque reduction assay [45,46]. The CC_50_ and IC_50_ values (Table 3) were determined by nonlinear regression of the concentration-response curves. As expected, B-NP did not show anti-herpetic activity against the HSV-1 at the tested concentration range (2.5–30 µg mL^−1^). The average IC_50_ (6.7 µg mL^−1^) and SI values (33.0) assayed for CQ were considerable larger than that for CQ-NP (4.3 µg mL^−1^, 15). All the three distinct parameters evaluated have demonstrated the superior anti-HSV-1 efficacy of CQ-NP.

**Figure 4:**
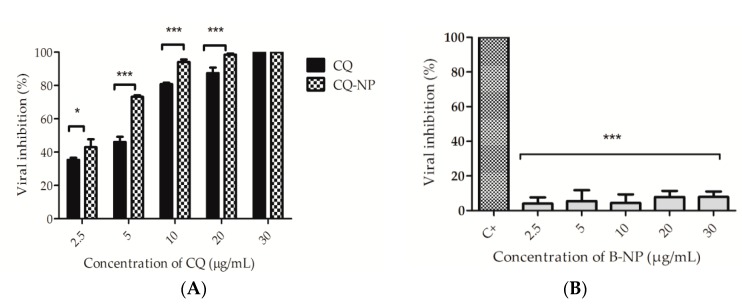
Antiviral activity of chloroquine (CQ), chloroquine loaded PLA nanoparticles (CQ-NP), and blank nanoparticles (B-NP). (**A**) Viral inhibition percentage after treatment for 48h with CQ and CQ-NP; and (**B**) viral inhibition percentage after treatment for 48h with B-NP. The positive control (C^+^) of all experiments was acyclovir (20 µg mL^−1^). Notes: The data were expressed as mean ± standard deviation (SD) (*n* = 3), (*): *p* < 0.05 and (***): *p* < 0.001. The experiments were analyzed by the Two-way ANOVA following by the Bonferroni’s test (**A**) and the One-way ANOVA followed by the Dunnett’s test (**B**).

Further experiments aiming to evaluate the anti-herpetic activity of the formulations were performed after virus adsorption. Acyclovir (20 µg), the first-line drug for the treatment of HSV infections, was used as positive control, whereas untreated infected cells were used as negative control. The treatment with different concentrations of samples for 48 h post-infection (p.i) showed a dose-dependent viral inhibition by both CQ and CQ-NP (*p* < 0.001) (Figure 4A). The results suggested that CQ showed the ability to inhibit 100% of viral replication at 30 µg mL^−1^, whereas, for CQ-NP, this effect was observed at 10 µg mL^−1^ (Figure 4A). At the concentration range from 5 to 20 µg mL^−1^, the CQ-NP had an inhibitory activity considerable higher than CQ with *p* < 0.001. (***). Moreover, a smaller difference *p* < 0.05 (*) was observed at the concentration of 2.5 µg mL^−1^. The treatment of Vero E6 infected cells with all tested concentrations of B-NP did not show significant activity against the virus (Figure 4B), once the inhibition of viral replication was significantly different from the positive control with *p* < 0.001(***).

## 4. Discussion

In this study, the feasibility of biodegradable PLA nanoparticles as a promising approach to improve antiviral activity of Chloroquine diphosphate (CQ) has been investigated. Small biodegradable nanoparticles have the ability to overcome biological barriers, such as the efflux cotransport proteins at cell membrane and until brain-blood barrier [26,29]. In this context, Vero E6 cells were infected with a common human pathogen, the herpes simplex virus type 1 (HSV-1). The antiviral activity of CQ has been reported in the literature [18,19,20,21,22,23,24]. However, its anti-HSV-1 activity was not yet been well described. Indeed, due to the similar characteristic with previous described virus infections studies using CQ, a promising effect against HSV-1 infection could be hypothesized using this drug. In addition, its encapsulation in the biodegradable and biocompatible NP could solve its non-specific targeting against intracellular pathogens [25]. 

The biocompatible and biodegradable poly(lactic acid) (PLA) was chosen for this purpose. However, this polymer is a hydrophobic polyester [29] and produce NP for encapsulating hydrophilic drugs, such as the CQ, consists of an interesting challenge [30,31]. Thus, two different methods of preparation of nanoparticles well applied for this class of polymer were carefully tested. The small size of the system and the drug encapsulation efficiency (EE) were selected as the main performance parameters. Physicochemical properties of the formulations produced by the nanoprecipitation method revealed that the drug/polymer ratio directly affected both particle size and EE% (Table 1). As expected, the particle size increased according to the polymer concentration in the organic phase. This effect generally improves the EE% of hydrophobic drugs [32,33,47,48]. In contrast to this fact, Formulations 1, 2, and 3 showed low EE%, which decrease with the polymer increment. Chloroquine Diphosphate (CQ) is a low-weight drug with multiple pKas, and solubility behavior dependent on pH. At this pH = 6.4, this hydrophilic compound is unable to favorable interaction with hydrophobic polyesters such as PLA. The increment of PLA leads to drug displacement from organic phase to aqueous phase, resulting in low entrapment efficiency [30,31,32,33,49].

The experimental results with formulations one-three provided important achievements about the relationship between the solubility behavior of CQ and its interaction with the PLA. Thus, the pH of the aqueous phase, initially controlled at pH = 6.4, was adjusted to an alkaline range to increase the non-ionized fraction of CQ. This strategy can potentially improve the EE%, mainly considering hydrophilic drugs [33]. The central drug/polymer ratio of 1:10 was chosen for this purpose. Alkalinizing the aqueous phase (pH = 11.0) with NaOH in the Formulation 4 made possible improve the average EE% from 8.4% to 11.4%. Alkalinizing aqueous phase with NaHCO_3_ for pH = 8.4 in Formulation 5 proved to be more efficient, inducing an average EE% of about 25%. Previous studies have reported that CQ degradation is accelerated in 0.1 M NaOH media, by a hydrolysis mechanism [50]. The hypothesis of drug precipitation in the aqueous phase, due to the strongest alkaline pH, should also be considered.

The dispersions prepared at pH = 6.4 showed negative zeta potential (−3.13 mV to −14.42 mV) due to the terminal acid carboxyl groups of PLA (Table 1) [51]. Two main stabilization mechanisms for colloidal suspensions should be considered. The electrostatic repulsion, in which adding ionic stabilizers, and the steric stabilization that can be achieved by non-ionic stabilizers into the medium. The formulations described in the present study are stabilized using poloxamer 188, a non-ionic and copolymer stabilizer agent. The steric stabilization is dominated by solvation effect [52]. Thus, pH changes or changes in drug/polymer ratio not necessary induces monotonically change in the zeta potential, as occurred in this approach (Table 1). This value slightly increased for Formulation 3 with the largest PLA content. When the PH increased for alkaline range (eight and eleven), the zeta potential remained negative, as expected.

**Figure 5:**
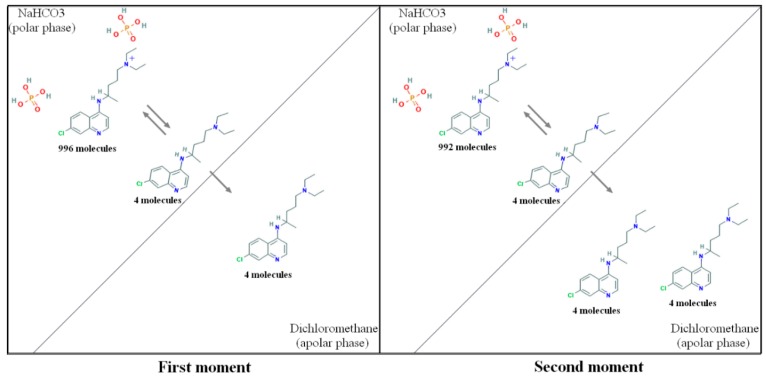
Suggested chloroquine partitioning scheme. At the first moment, a theoretical maximum ratio of ionized chloroquine molecules occurs in the polar phase following by a gradually partitioning to the organic phase at the second moment. This transport follows until the exhaustion with the total partition of the drug to the organic apolar phase.

The selected parameters for the emulsification-solvent evaporation method induced larger particles compared to that produced by the nanoprecipitation technique (Table 2). NP have shown negative zeta potential and narrow particle size (PdI = 0.3) with mean diameter <300 nm. The best results using the nanoprecipitation method were reached using the CQ:PLA ratio of 1:5 *w*/*w* and 0.5 M NaHCO_3_ aqueous solution (pH = 8.4). These work conditions were repeated for the emulsification-solvent evaporation method, with an excellent average EE% level of about 64.1%. This fact corroborated with the successful partition of CQ-fb to the organic phase and the importance of the alkaline aqueous phase to maintain the drug and polymer in the droplets of the emulsion. The pH of the aqueous phase (0.5 M NaHCO_3_, pH = 8.4) assured a maximum of non-ionized of chloroquine’s amine groups (Pka_1_ = 8.4, Pka_2_ = 10.8), compared to the previous tested aqueous phase (pH = 6.4). At contact with the organic immiscible solvent (DCM), the phase equilibrium between the ionized and the non-ionized species of CQ in the aqueous phase changes constantly, with the partition of non-ionized specie of the drug to the interface, and finally to the organic solvent. The displacement of non-ionized molecules of drug occurs until its exhaustion and transport of the total amount to the organic phase, as suggested, for example, in the Figure 5. The mass ratio maintained between the two phases could be explained by the principle of mass conservation.

The Formulation 6 was selected for further experiments. The AFM images (Figure 1) suggested spherical NP with slightly smooth surface. It was also possible to observe NP in the range of 200 nm, corroborating to the DLS experiments. Hoo et al. (2008) reported some differences among AFM and DLS experiments, mainly due to the differences in sample preparing procedures, which can induce some particle agglomeration [53]. 

The Formulation 6 was able to generate the desired slow drug release, corroborating with the drug-loading experiments (Figure 2). Considering the emulsification-solvent evaporation method, the EE% level superior to 60% is considered excellent, which induced a slow drug release profile adjusted by the parabolic diffusion mathematical model (*r* ≥ 0.95) (Table 2). This fact suggested a diffusion-controlled transport of CQ from the polymeric matrix of NP [43]. In addition, it is important to note that, on the best of our knowledge, no data in the literature describes the slow chloroquine release from PLA nanoparticles. Previous studies have shown a release of 52.40% of hydroxyl chloroquine sulphate from Eudragit^®^ RL-100 NP (EE% = 63.14%) at 8 h [54]. The encapsulation of chloroquine diphosphate was described in chitosan NP prepared by ionic gelation technique (EE% = 59.5%), which induced the release of 40% of the drug at 24 h [55]. In the present study, the amount of drug released at 10 h was about 40%, which is much slower than the previously cited studies. In this approach, the residual amount of non-ionized specie of CQ loaded into PLA nanoparticles seems to be strongly interacting with PLA matrix. The parabolic model fitted the experimental data much better (*r* = 0.95) (Figure 2E), which suggested a drug release controlled by diffusion at the first moment. However, the erosion of polymeric matrix of particles should also be considered for release of residual entrapped drug, which could occur in a second moment [44,48,49,51]. 

This ability of colloidal nanocarriers to preserve the entrapped drug into the polymeric matrix is a promising characteristic for drug targeting intended for specific affected cell or tissue, mainly considering the internalization of nanoparticles by endocytosis [43,56,57]. 

The assessed physicochemical and feasible properties of the Formulation 6 suggested an interesting and promising PLA NP system for CQ targeting. Thus, studies for evaluating its cytotoxicity and its anti-HSV-1 activity were performed. The cell viability experiments demonstrate the absence of cytotoxic effect of blank PLA NP (B-NP) in the tested concentration range (7.81–500 µg mL^−1^) for 24 h (Figure 3A) and 48h of incubation (Figure 3B). In addition, it was possible to suggest that PLA NP improved CQ uptake, because CQ-NP have shown cell viability levels lower than that observed for free CQ drug at the same tested concentration range (Figure 3C). The CC_50_ identified for CQ (222.6 µg mL^−1^) and CQ-NP (67.9 µg mL^−1^) (Table 3) corroborated with this fact. The biocompatible (non-cytotoxic) concentration range of CQ from NP was selected to be used in the following experiment with HSV-1-infected cells. The positive control, acyclovir, was used in this experiment at non-cytotoxic concentration (20 µg mL^−1^).

The first-line of treatment for HSV infection consists in the use of acyclic analogue of guanosine and its derivatives. While these drugs have distinct metabolic pathways, they all act by blocking the synthesis of viral DNA, inhibiting DNA polymerase [58]. Three phosphorylation steps are required for the acyclovir action. The first phosphorylation is performed by the viral thymidine kinase (TK) encoded by the UL23 gene, while subsequent phosphorylation steps are performed by the host cell kinases [59]. Currently, there are some strains of HSV-1 resistant to acyclovir, in which the presence of mutations in UL23 and/or UL30 viral genes responsible for encoding TK and DNA polymerase, were detected [60]. The emergence of new strains resistant to conventional drugs highlight the need for new effective antiviral therapies with novel mechanisms of action [61,62]. Thus, new anti-herpetic drugs with different mechanism of action should be achieved. 

In this context, this work focused on the role of CQ, which is a highlighted lysosomotropic drug among the molecules with proved antiviral activity [18,19,20,21,22,23,24,63]. The CQ has shown pronounced antiviral action against different viruses [64]. The study reported by Koyama and Uchida (1984) demonstrated that the multiplication of the HSV-1 strain HF in Vero cells was inhibited by ammonium chloride and chloroquine. It was also observed that the maturation of the intracellular virus was prevented immediately after the addition of weak bases in the late stage of infection, indicating that the site of inhibition by weak bases is one step in the maturation process of HSV-1 [34]. This present study showed that Vero E6 cells infected with HSV-1 and treated with CQ and CQ-NP at 30 µg mL^−1^ have 100% of viral replication inhibition after 48 h (Figure 4A). At the concentration range from 2.5 to 20 µg mL^−1^, the PLA NP significantly improved the anti-HSV-1 of CQ (Figure 4A). The CQ-NP induced an IC_50_ considerable lesser than that identified for CQ (Table 3). In addition, the CQ-NP (at 10 µg mL^−1^ and 20 µg mL^−1^) induced similar HSV-1 viral inhibition than that observed for the positive control, acyclovir, at 20 µg mL^−1^ (Figure 4B). 

It is well reported that polymeric nanoparticles change drug bioavailability and biodistribution, reducing side effects, and improving drug efficacy [65]. Previous studies have also demonstrated that these nanoparticles are biocompatible systems, using a cell culture approach [66,67]. The mechanism of CQ action in infected cells is still not fully solved, but some studies have proposed the changes in lysosomal pH, with inhibition of viral packaging and maturation through the trans-Golgi network [38,68]. Thus, considering the reported resistant strains of HSV-1 to acyclovir [60], CQ-loaded PLA nanoparticles system can be suggested as an interesting and promising approach against this viral infection.

## 5. Conclusions

In this study, we found that nanoprecipitation was not the best method to encapsulate the hydrophilic CQ drugs when compared to emulsification-solvent evaporation. However, as shown in our experiments, we tried different strategies and considered important parameters to improve drug loading. Biocompatible PLA nanoparticles improved the anti-HSV-1 activity of chloroquine. The data have demonstrated an efficient nanoencapsulation method, using emulsification-solvent evaporation, able to produce sub-300nm NP, with high EE% of the hydrophilic drug, and able to induce slow CQ release. The experimental results also revealed that CQ-NP induced similar HSV-1 inhibition that observed for the first line of the anti-HSV-1 drug acyclovir. In addition, it could be suggested that PLA NP improved CQ uptake by infected Vero E6 Cell, consisting in a promising approach to solve the drug limitations to overcome biological barriers and targeting affected cells. Further studies are required to corroborate with this hypothesis using animal models or more complex tests to assess possible strategies for functionalizing NP. 

## Figures and Tables

**Table 1:** Physicochemical properties of blank nanoparticles (B-NP) and respective CQ-NP produced by the nanoprecipitation method.

Formulation	CQ ^1^:PLA ^2^ Ratio	AP ^3^ pH	Size (nm)	PdI ^4^	ZP ^5^ (mV)	EE ^6^ (%)
B-NP	0	6.4	106.2 ± 2.5	0.157 ± 0.03	−7.95 ± 3.2	
1	1:5	6.4	173.5 ± 8.5	0.113 ± 0.04	−3.13 ± 3.2	10.6 ± 1.3
2	1:10	6.4	189.1 ± 6.5	0.080 ± 0.03	−6.85 ± 5.3	8.4 ± 2.6
3	1:15	6.4	226.4 ± 9.2	0.073 ± 0.03	−14.42 ± 2.1	3.4 ± 1.4
B-NP	0	11.0	114.5 ± 6.0	0.089 ± 0.02	−1.91 ± 0.8	
4	1:10	11.0	200.6 ± 11.4	0.069 ± 0.01	−18.15 ± 3.3	11.4 ± 2.0
B-NP	0	8.4	118.6 ± 6.0	0.046 ± 0.01	−11.63 ± 3.0	
5	1:10	8.4	231.4 ± 11.5	0.096 ± 0.02	−5.68 ± 4.1	25.0 ± 1.6

Note: Data are expressed as mean ± standard deviation (*n* = 3). ^1^ CQ, chloroquine diphosphate; ^2^ PLA, poly(lactic acid); ^3^ AP, aqueous phase; ^4^ PdI, polydispersity index; ^5^ ZP, zeta potential; ^6^ EE%, encapsulation efficiency.

**Table 2:** Physicochemical properties of B-NP and CQ-NP (formulation 6) produced by the emulsification-solvent evaporation method and their fitting parameters of different kinetic models applied for the in vitro drug release experiments.

Formulation	Physicochemical Properties				Kinetic Models [*k* (R)]			
	Size (nm)	PdI ^1^	ZP ^2^ (mV)	EE ^3^ (%)	First Order	Bhaskar	Freundlich	Parabolic
B-NP	283.9 ± 53.2	0.27 ± 0.05	−25.4 ± 11.6	-	-	-	-	-
6	297.3 ± 26.1	0.30 ± 0.03	−20.0 ± 12.0	64.1 ± 5.0	0.004 h^−1^ (0.88)	0.26 h^0.65^ (0.94)	63.99 (0.94)	2.25 h^−0.5^ (0.95)

Note: Data are expressed as mean ± standard deviation (*n* = 3). ^1^ PdI, polydispersity index; ^2^ ZP, zeta potential; ^3^ EE, encapsulation efficiency; values of drug release rate constant (*k*); correlation coefficient (*R*).

**Table 3:** Cytotoxicity and parameters of anti-HSV-1 activity evaluated for chloroquine (CQ), blank PLA nanoparticles (B-NP) and chloroquine-loaded PLA nanoparticles (CQ-NP) in Vero E6 cells.

Samples (Treatment 48 h)	CC_50_^1^ (µg mL^−1^)	IC_50_^2^ (µg mL^−1^)	SI ^3^
B-NP	>500	^4^ N.A	-
CQ	222.6 ± 5.4	6.7 ± 0.6	33.0
CQ-NP	67.9 ± 2.1	4.3 ± 1.4	15.6

Note: Data are expressed as mean ± standard deviation (SD) from three independent experiments with each treatment performed in triplicate. ^1^ CC_50_ = concentration that was cytotoxic for 50% of the Vero cells; ^2^ IC_50_ = concentration that inhibited viral replication by 50% in the post-treatment conditions; ^3^ SI = selectivity index, calculated from the ratio of CC_50_ and IC_50_; ^4^ N.A: No activity.
